# Comparison of separation methods for immunomodulatory extracellular vesicles from helminths

**DOI:** 10.1002/jex2.41

**Published:** 2022-05-03

**Authors:** Anne Borup, Anders T. Boysen, Andrea Ridolfi, Marco Brucale, Francesco Valle, Lucia Paolini, Paolo Bergese, Peter Nejsum

**Affiliations:** ^1^ Department of Clinical Medicine Aarhus University Aarhus Denmark; ^2^ Consorzio Interuniversitario per lo Sviluppo dei Sistemi a Grande Interfase (CSGI) University of Florence Florence Italy; ^3^ Consiglio Nazionale delle Ricerche (CNR) Istituto per lo Studio dei Materiali Nanostrutturati (ISMN) University of Bologna Bologna Italy; ^4^ Department of Chemistry University of Florence Florence Italy; ^5^ Department of Molecular and Translational Medicine University of Brescia Brescia Italy; ^6^ Consiglio Nazionale delle Ricerche (CNR) Institute for Research and Biomedical Innovation (IRIB) University of Palermo Palermo Italy

**Keywords:** Ascaris, extracellular vesicles, helminth, host‐parasite interaction, immune modulation, separation methods

## Abstract

Helminths survive within their host by secreting immunomodulatory compounds, which hold therapeutic potential for inflammatory conditions. Helminth‐derived extracellular vesicles (EVs) are one such component proposed to possess immunomodulatory activities. Due to the recent discovery of helminth EVs, standardised protocols for EV separation are lacking. Excretory/secretory products of the porcine helminth, *Ascaris suum*, were used to compare three EV separation methods: Size exclusion chromatography (SEC), ultracentrifugation (UC) and a combination of the two. Their performance was evaluated by EV yield, sample purity and the ability of EVs to suppress lipopolysaccharide (LPS)‐induced inflammation in vitro. We found that all three separation methods successfully separated helminth EVs with a similar EV yield. Functional studies showed that EVs from all three methods reduced LPS‐induced levels of tumour necrosis factor (TNF‐α) in a dose‐dependent manner. Overall, the three separation methods showed similar performance, however, the combination of UC+SEC presented with slightly higher purity than either method alone.

## 1 INTRODUCTION

Extracellular vesicles (EVs) are membrane‐enclosed nanoparticles containing a variety of bioactive molecules such as proteins, nucleotides and lipids (Jeppesen et al., 2019). They are released through various pathways by numerous cell types making them present in different body fluids from where they are internalised by recipient cells (Doyle & Wang, 2019). Traditionally, mammalian EVs are categorised based on their mechanism of biogenesis and release as ‘exosomes’ or ‘microvesicles’. Exosomes (30–100 nm in diameter) originate through an endosomal pathway by creating multivesicular bodies whilst microvesicles (50–1000 nm in diameter) arise by outward budding of the plasma membrane (Mathieu et al., 2019; Van Niel et al., 2018). The heterogeneous nature of EVs complicates the discrimination of subtypes by currently used separation techniques. However, refinement of these methods has paved the way to the recent discoveries of novel membrane‐less extracellular particles such as exomeres (<50 nm) and supermeres (<50 nm) that both differ in size and composition from other EVs (Zhang et al., 2018, 2021). According to the most recent guidelines from the International Society of Extracellular Vesicles (ISEV), the most acceptable subdivisions are ‘small EVs’ (<200 nm) and ‘large EVs’ (>200 nm) (Théry et al., 2018). However, with the rapidly expanding EV field and the addition of new terms, such as extracellular particles, this terminology will evolve in the future. Due to the lack of absolute discrimination of subtypes, we will here only use the generic term ‘extracellular vesicles’.

The importance of EVs is highlighted by their central role in intercellular communication (Wiklander et al., 2019). Recent discoveries have uncovered a crucial role for EVs in communication between organisms and cross‐species, such as host‐parasite interaction. Two species of parasitic worms (helminths) were first found to release EVs as part of their excretory/secretory products (ESPs) by Marcilla et al. (2012). As reviewed elsewhere, helminth‐derived EVs can modulate and suppress the host immune response and are therefore crucial for host‐parasite interactions but may also hold therapeutic potential (Drurey & Maizels, 2021; Mardahl et al., 2019; Sánchez‐López et al., 2021; Zakeri et al., 2018). In assorted mouse models of colitis, helminth EVs show preventive and anti‐inflammatory effects. These findings underline the therapeutic potential helminth‐derived EVs hold for autoimmune diseases and allergic disorders. However, the exploration of the role of helminth EVs in host‐parasite interactions and their therapeutic potential is hampered by the lack of standardised guidelines for separating EVs from non‐vesicular components of the ESPs. This problem has been highlighted and investigated for mammalian samples including plasma and conditioned cell culture media (Askeland et al., 2020; Brennan et al., 2020; Dong et al., 2020; Takov et al., 2019) where the optimal method was largely dependent on the selection of downstream analysis. ESPs as a source of EVs is far less investigated (Royo et al., 2020). As components of ESPs are different from human fluids and culture media, the knowledge obtained from mammalian EVs may not be directly applicable to research on helminth EVs. Furthermore, although the biogenesis of helminth EVs includes some of the same machinery as mammalian EVs, other unique processes and proteins are also involved (Bennett et al., 2020). To date, only one study has compared separation methods for helminth EVs (Davis et al., 2019).

There is a broad spectrum of techniques to separate EVs that utilise various characteristics of EVs. This creates different categories of methods such as centrifugation‐based separation, column chromatography, filtration techniques, polymer precipitation and affinity purification (Carnino et al., 2019; Liangsupree et al., 2021; Ramirez et al., 2018; Sidhom et al., 2020). Briefly, a centrifugation‐based approach was the original EV separation method and often consists of several centrifugation steps at increasing centrifugal force and for small EVs ending with one or more ultracentrifugation (UC) steps (100,000–120,000 × *g*). EVs are separated primarily based on their particle density, but size and shape also affect the sedimentation rate. Density gradient centrifugation is a variant of UC that applies a gradient of, for example, sucrose to take further advantage of EV density. For column chromatography, size exclusion chromatography (SEC) is predominately used. The column is packed with porous particles that allow EVs to be eluted separately from other components, such as proteins, based on size differences. Filtration techniques also utilise the size of the EVs and separate EVs by passing them through a filter that retains particles larger than the molecular weight cut off the selected filter (Busatto et al., 2018). For precipitation, hydrophilic polymers are often used to bind the water molecules and thereby precipitate the EVs (Rider et al., 2016). Finally, EVs can be separated by affinity capture by using immobilised antibodies or peptides that bind EV‐specific antigens (Koliha et al., 2016; Nakai et al., 2016). This highlights the variety of methods used for EV separation and the continuous development of new technologies. A major hurdle for EV separation is contaminating compounds that can affect the downstream analysis and vary depending on the EV source (Ramirez et al., 2018). Common contaminating factors in mammalian EV preparation include serum albumin and lipoproteins. Helminth EVs can be contaminated with residues from the host or bacteria, parts of the worms tegument or cuticle and worm or egg debris (Siles‐Lucas et al., 2017). However, identification of these compounds can be troublesome and some of them may also be naturally present in EVs. In order to reduce the level of contaminating factors, a combination of two separation techniques, which utilise different properties of the EVs, have been recommended for mammalian EVs. Based on a survey from 2019, it was established that the most commonly used separation method was UC, however, usage of SEC was on the rise (Royo et al., 2020; Sotillo et al., 2020). Therefore, these two methods alone or in combination are compared in this paper.

Optimal separation methods are able to obtain an EV preparation with low contamination of non‐EV related components, such as non‐vesicular proteins and lipids, while recovering a high proportion of EVs that are identified by state‐of‐art methods (Dong et al., 2020; Théry et al., 2018). Research in helminth EVs is challenged by a lack of traditional EV markers such as are used for Western blotting of mammalian EVs (e.g., CD63, CD81, and ALIX) and therefore problems in determining what are considered contaminating non‐EV components (Ramirez et al., 2018). Some markers have been identified for EVs released by trematodes or cestodes, however, as they are from a different phylum and therefore biologically different from the nematodes it is difficult to translate the findings (Sotillo et al., 2020). In the absence of markers, the degree of contamination can be estimated by different approaches such as the colourimetric nanoplasmonic (CONAN) assay, which evaluates the amount of non‐EV protein, and atomic force microscopy (AFM) where identified non‐vesicular structures can be considered as contaminating (Ridolfi et al., 2020; Zendrini et al., 2020). Another parameter for an optimal separation method is its ability to preserve the functionality of the EVs as evaluated by different in vitro experiments.

**FIGURE 1:**
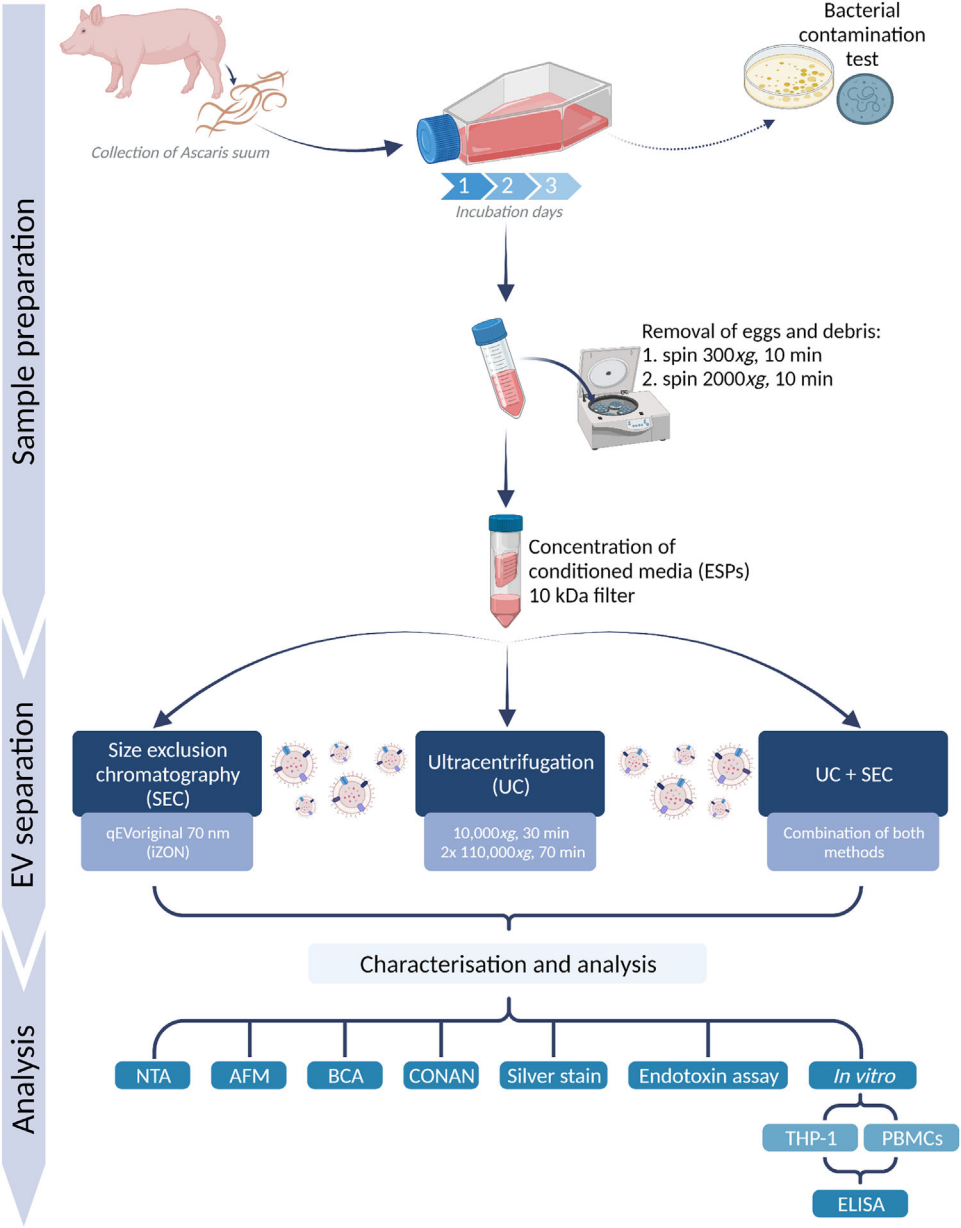
Schematic summary of the study design. *Ascaris suum* were collected, washed and incubated for three days in culture medium supplemented with antibiotics/antimycotics. The conditioned media (excretory/secretory products [ESPs]) was tested for bacterial contamination (agar plates and mycoplasma activity) before being cleared from eggs and debris by centrifugation. Before EV separation, the ESPs were concentrated on 10 kDa filters. EVs were separated by either size exclusion chromatography (SEC), ultracentrifugation (UC) or a combination of both. These methods were compared based on EV characterisations analysed using nanoparticle tracking analysis (NTA), atomic force microscopy (AFM), BCA, COlorimetric NANoplasmonic (CONAN) assay, silver stain, endotoxin assay and functional studies using a cell line (THP‐1) and human peripheral blood mononuclear cells (PBMCs). Created with BioRender.com

In this study, we compared three distinct separation procedures (UC, SEC, and UC followed by SEC) for their efficacy in separating EVs released by the porcine helminth *Ascaris suum* (Figure 1). We show that combining two separation methods (UC+SEC) results in the lowest amount of non‐EV particles without a significant loss in EV concentration. Furthermore, we provide first insights into the immunomodulatory function of EVs derived from adult *A. suum* ESPs, which contribute to our understanding of host‐parasite interactions.

## 2 MATERIALS AND METHODS

**Resource availability**

Lead contact

Further information and requests for resources and reagents should be directed to the lead contact, Peter Nejsum (pn@clin.au.dk).

Materials availability

This research did not generate new unique reagents.

Data and code availability

The paper does not include any original code.

Any need for additional information in order to reanalyse the reported data is available per request from the lead contact.

### 2.1 Culture of helminths

Adult *Ascaris suum* were collected from naturally infected pigs at an abattoir (DAT‐Schaub A/S, Herning, Denmark). After an initial wash in 37°C saline (0.9% NaCl [Sigma‐Aldrich, St. Louis, MO, USA]) worms were immediately transported to the laboratory. Five *A. suum* were distributed at a ratio of 3:2 (female:male) per T175 flask. Worms were incubated with saline (0.9% NaCl) and an optimised Antibiotic‐Antimycotic mixture (200 μg/ml Streptomycin, 200 U/ml Penicillin G, 1.25 μg/ml Amphotericin B from *Streptomyces sp*. and 10 μg/ml Ciprofloxacin) (all reagents from Sigma‐Aldrich, St. Louis, MO, USA) for a total of four cycles of 15 min followed by three times 1 h with a buffer exchange between cycles. All incubations were performed at 37°C. Following the washing steps, worms were incubated in 175 ml RPMI‐1640 + antibiotic‐antimycotic mixture (100 μg/ml Streptomycin, 100 U/ml Penicillin G, 0.63 μg/ml Amphotericin B and 5 μg/ml Ciprofloxacin) at 37°C and 5% CO_2_ under sterile conditions for a total of 3 days. Conditioned media containing the ESPs were collected and exchanged daily by decanting. ESPs were checked daily for bacterial and mycoplasma contamination. Culture media was distributed to Luria Bertani agar plates to inspect aerobic bacterial growth after 24 h, while mycoplasma was tested using the MycoAlert Mycoplasma Detection kit (Lonza Group AG, Gampel, Switzerland). The level of endotoxin in ESPs and EV preparations was evaluated using the HEK‐Blue™ LPS Detection Kit 2 (InvivoGen, Toulouse, France) following the manufacturer's protocol. Preliminary experiments concluded that the antibiotic/antimycotic concentration and composition used for washing and incubation of the worms were sufficient to avoid bacterial contamination.

The collected ESPs were subjected to differential centrifugation at 300 × *g* for 10 min followed by 2000 × *g* for 10 min to remove helminth eggs and debris. The cleared ESPs were stored at −80°C until further processing. EVs separated from different incubation days showed no significant difference in functional properties (Figure S1). Therefore, ESPs from the three incubation days were pooled to a final volume of 270 ml and concentrated to 500 μl using an Amicon® Ultra‐15 Centrifugal Filter Unit 10 kDa cut‐off (Merck Life Science, Burlington, MA, USA). The concentrated ESPs are referred to as *ESPs* throughout this study unless stated otherwise.

### 2.2 Separation of EVs from *Ascaris suum* ESPs

#### 2.2.1 Size exclusion chromatography (SEC)

EVs were separated using qEVoriginal/70 nm columns (*SEC*) (iZON Science Ltd). The column was equilibrated with 10 ml of PBS (0.22 μm filtered) (Biowest, Nuaillé, France). The ESPs (500 μl) was added to the column followed by elution with PBS (0.22 μm filtered) and fractions of 500 μl collected. Due to the difference in characteristics between mammalian EVs, which the SEC column is designed for, and helminth EVs, the particle and protein concentration of eluted fractions 1–24 were evaluated. Fractions 1–6 represent the column void volume, fractions 7–10 contained EVs, fractions 11–15 contained a combination of EVs and proteins, while fractions 16–24 contained eluted soluble non‐EV proteins (Figure S4). The EV‐containing fractions 7–10 were pooled and stored at −80°C until use.

#### 2.2.2 Ultracentrifugation (UC)

EVs were separated using differential centrifugation with the last step being UC modified from Kowal et al. (2016). First, 500 μl of the ESPs were diluted four times with PBS (0.22 μm filtered) to a final volume of 2 ml. The sample was centrifuged at 10,000 *× g* for 30 min at 20°C (Heraeus Biofuge Pico, Hanau, Germany). The supernatant was transferred to polycarbonate ultracentrifuge tubes with cap assembly (Beckman Coulter, Brea, CA, USA) and further diluted five times with PBS to a final volume of 10 ml. The sample was centrifuged at 110,000 *× g* for 70 min at 4°C (40,800 RPM TI‐50 rotor) (Beckman Coulter, Brea, CA, USA). The pellet was resuspended in 10 ml PBS and centrifuged at 110,000 × *g* for 70 min at 4°C and the supernatant discarded. The final pellet was resuspended vigorously in 2 ml PBS (0.22 μm filtered) and stored at −80°C until needed. For the combination with the SEC procedure, the pellet was resuspended in 500 μl PBS (0.22 μm filtered) and loaded to the qEVoriginal/70 nm columns (iZON Science Ltd) with the collection of fractions 7–10 as previously described.

### 2.3 Characterisation of EVs

#### 2.3.1 Atomic force microscopy (AFM)

EVs were analysed using atomic force microscopy (AFM)‐based high‐throughput nanomechanical screening method as described elsewhere (Ridolfi et al., 2020). Controlled surface charge density substrates were prepared as follows. Borosilicate glass coverslips (Menzel Gläser, Braunschweig, Germany) were cleaned for 2 h in 3:1 (v/v) H_2_SO_4_/H_2_O_2_, rinsed in ultrapure water, then sonicated in a sonicator bath (Elmasonic S30H) for 30 min in acetone, 30 min in isopropanol and 30 min in ultrapure water (Millipore Simplicity UV). Glass slides were then exposed for 5 min to air plasma (Pelco Easiglow), incubated for 30 min in a 0.01 mg/ml poly‐L‐lysine (PLL) solution in 100 mM borate buffer (pH 8.33) at room temperature (RT), thoroughly rinsed with ultrapure water and dried with nitrogen. EV samples were diluted 10× in ultrapure water then deposited on a freshly prepared PLL‐functionalised glass slide and left to adsorb for 30 minutes at 4°C, then inserted in the AFM fluid cell (see below) without further rinsing.

All AFM images were recorded on a Multimode 8 microscope (Bruker, USA) equipped with a Nanoscope V controller and a type JV piezo scanner. Samples were measured in a sealed fluid cell filled with ultrapure water using ScanAsyst Fluid+ probes (Bruker, USA). Raw images were processed with Gwyddion v2.58 (Nečas & Klapetek, 2012) for background subtraction. Quantitative morphometry of EVs was performed as described elsewhere (Ridolfi et al., 2020).

#### 2.3.2 Nanoparticle tracking analysis (NTA)

The concentration and size distribution of particles in the samples were determined by nanoparticle tracking analysis (NTA) using a Nanosight NS300 system (Malvern Panalytical, Malvern, UK) coupled with a Nanosight syringe pump (Malvern Panalytical, Malvern, UK). The system was equipped with a 405 nm laser and a sCMOS camera. The system was validated before sample analysis by analysing polystyrene beads of 97 nm (Bachurski et al., 2019). Samples were diluted to a final volume of 1 ml with PBS (0.22 μm filtered) to obtain the optimal particle per frame value (20–100 particles/frame) (Bachurski et al., 2019; Gardiner et al., 2013). The samples were infused into the system at a constant flow of 20 μl/min using a 1 ml syringe mounted to a syringe pump and a constant temperature of 25°C. Particles were detected at a camera level of 15 and recorded for 5 *×* 60 seconds. The recordings were processed by the Nanosight 3.4 software (Malvern Panalytical, Malvern, UK) using a detection threshold of 5.

The 0.22 μm filtered PBS used for all EV separations and downstream analysis was evaluated for particle contamination using NTA. The particle concentration was <2 × 10^6^ particles/ml (particles/frame = <2) concluding that the PBS contributes with a negligible particle contamination.

#### 2.3.3 Bicinchoninic acid (BCA) assay

The concentration of proteins in the samples was determined using the Pierce™ BCA Protein Assay kit (Thermo Fisher Scientific, Waltham, MA, USA) according to the manufacturer's protocol using 10 μl of the sample in a 96‐well plate. Samples were measured in triplicate. Reactions were incubated for 15–30 min at 37°C. Absorbance was read at 562 nm and calculations of the concentrations were performed using a four‐parameter logistic curve.

Based on the suggestion by Webber and Clayton (2013), the purity of the preparations was estimated using the particle/protein ratio following this equation:

(1) $$Purity\;\left(Particles/Protein\right)=\;\frac{Particles/ml}{\mu g/ml}$$

#### 2.3.4 COlorimetric NANoplasmonic (CONAN) method

COlorimetric NANoplasmonic (CONAN) assay was applied to evaluate preparations’ purity from exogenous protein contaminants as described elsewhere (Zendrini et al., 2020). The CONAN assay exploits the nanoplasmonic properties of colloidal gold nanoparticles (AuNPs) and their peculiar interaction with proteins and lipid bilayers (Maiolo et al., 2015). The assay consists of an aqueous solution of bare gold nanoparticles (synthesised through Turkevich's citrate reduction method; Turkevich et al., 1951) at a 6 nM concentration. When mixed with pure EV preparations, the AuNPs will cluster on the EV membrane, whereas in EV preparations that contain high amounts of non‐EV protein contaminants the AuNPs will preferentially cluster together and not adhere to the EV membrane. Upon clustering to the EV membrane, their localised surface plasmon resonance (LSPR) is broadened, resulting in a colour change of the AuNP solution from red to blue. This can be accurately monitored through UV−Vis spectroscopy. This colour change is directly related to the purity grade of the added EV preparation and can be quantified by calculating the aggregation index (AI), defined as the ratio between the absorbance intensity at the LSPR peak and the absorbance intensity at 650 and 850 nm. This AI can also be expressed as a percentage (AI%) of the ratio between the AI for the preparations and the AI of an AuNPs solution. An AI% below 20% indicates a pure solution.

The experiment was conducted by taking 2 μl of SEC, UC, and UC+SEC preparations, which were diluted by 1:1, 1:3, and 1:5 with Milli‐Q H_2_O to a final volume of 25 μl. To each solution, 50 μl of AuNP 6 nM was added. The mixes were incubated for 30 minutes at RT and subsequently the absorbance at 650 and 850 nm were measured using a UV‐Vis spectrophotometer.

#### 2.3.5 Silver stain

Samples were lysed, reduced and denatured with the addition of Laemmli buffer (Bio‐rad Laboratories Inc., Hercules, CA, USA), 0.1 M DTT (Sigma‐Aldrich, St. Louis, MO, USA) and incubated at 95°C for 10 min. Samples were then run on a Novex 4–12% Tris–Glycine Plus Midi Protein precast gel (Invitrogen, Carlsbad, CA, USA) and silver‐stained using Pierce™ Silver Stain kit (Thermo Fisher Scientific, MA, USA) following manufactures protocol. The developed gel was visualised using Chemidoc Imaging System (Bio‐rad Laboratories Inc., Hercules, CA, USA).

### 2.4 Functional analysis of the helminth‐derived EVs

#### 2.4.1 Cell culture

Human monocyte cell line, THP‐1 cells (ATTC, Manassas, VA, USA) (at passage 10), was seeded with a density of 100,000 cells per well in a TC Plate 96 Well (Cell+ with a flat base) (Sarstedt, Nümbrecht, Germany) in complete RPMI‐1640 (Biowest, Nauillé, France) with 10% heat‐inactivated foetal bovine serum (HI‐FBS) (Biowest, Nauillé, France). To differentiate the cells into macrophages, 100 nM phorbol‐12‐myristate‐13‐acetate (PMA) (Sigma‐Aldrich, St. Louis, MO, USA) was added for 24 h (37°C and 5% CO_2_). Subsequently, the cells were rested for 24 h in DMEM supplemented with 10% HI‐FBS (37°C and 5% CO_2_). Macrophages were stimulated with five different concentrations of *A. suum* particles (1000, 3500, 10,000, 30,000, and 150,000 particles per cell based on NTA) diluted in DMEM without supplements. Macrophages were stimulated with EVs for 30 min (37°C and 5% CO_2_) followed by ±10 ng/ml lipopolysaccharide (LPS) from *Escherichia coli* O26:B6 (Sigma‐Aldrich, St. Louis, MO, USA) stimulation and incubated for 24 h (37°C and 5% CO_2_). Experimental setup included positive (cells stimulated with 10 ng/ml LPS) and negative (unstimulated cells) controls. Each setup was conducted in triplicate.

Peripheral blood mononuclear cells (PBMCs) were isolated from human buffy coats from three healthy anonymous donors from the Danish blood bank at Aarhus University Hospital. PBMCs were isolated by density gradient centrifugation using SepMate‐50 tubes (STEMCELL Technologies, Vancouver, Canada) with Ficoll‐Paque PLUS (GE Healthcare, Uppsala, Sweden). Briefly, 50 ml of the buffy coat was diluted with 2% HI‐FBS in PBS (FBS+PBS) in a 1:1 (vol./vol.) ratio. Of this mixture, 30 ml was gently added to the SepMate‐50 tubes with 15 ml Ficoll‐Paque PLUS and centrifuged at 1200 *× g* for 10 min at RT. The supernatant was collected by decanting into 50 ml Falcon tubes following the addition of FBS+PBS to a total volume of 50 ml. The tube was centrifuged at 500 *× g* for 10 min at RT. The pellet was resuspended in 50 ml FBS+PBS and centrifuged at 500 *× g* for 10 min at RT. The pellet was resuspended in 1 ml FBS+PBS and cryopreserved with 20% Dimethyl Sulfoxide in liquid nitrogen until in vitro experiments.

The human PBMCs from the three donors were quickly thawed in pre‐heated PRMI‐1640 with 10% HI‐FBS and centrifuged at 500 *× g* for 5 min at RT. Cells were counted using the CASY cell counter (Cambridge Bioscience, Cambridge, UK) and 100,000 cells/well were seeded with RPMI‐1640 supplemented with 10% HI‐FBS to be incubated overnight (37°C and 5% CO_2_). The cells were stimulated with four different numbers of *A. suum* EVs (1000–30,000 particles per cell based on NTA; the 150,000 particles/cell used on THP‐cells were excluded for the more sensitive PMBCs), for 30 min before adding 10 ng/ml LPS from *E. coli*. Experimental setup included positive (cells stimulated with 10 ng/ml LPS) and negative (unstimulated cells) controls. Cells were incubated for 24 h (37°C and 5% CO_2_) followed by a centrifugation step of 500 *× g* for 5 min and supernatant collection for viability tests and quantification of cytokines. Each setup was conducted in duplicate.

#### 2.4.2 Cell viability

For both cell types, the viability after stimulation was assessed by CyQUANT™ LDH Cytotoxicity Assay (Invitrogen, Carlsbad, CA, USA) following manufacturer's protocol. Briefly, 50 μl of the supernatants were mixed with 50 μl of Reaction Mixture from the kit in a 96‐well plate. As a positive control of cell death, lysed cells were included. The plate was incubated for 30 min prior to absorbance measurement at 490 and 680 nm using the FLUOstar Omega microplate reader (BMG Labtech, Ortenberg, Germany).

#### 2.4.3 Quantification of cytokines

The concentration of cytokines was evaluated by DuoSet ELISA for tumour necrosis factor‐alpha (TNF‐α), IL‐6 and IL‐1β (all are from R&D systems, Minneapolis, MN, USA) in accordance with the manufactures instructions using Nunc MaxiSorp™ flat‐bottom plates (Thermo Fisher Scientific, MA, USA). The plates were developed using streptavidin‐HRP (R&D systems, Minneapolis, MN, USA) and TMB X‐tra (Kementec Solutions A/S, Taastrup, Denmark). The reaction was stopped by addition of 0.5 M sulphuric acid and absorbances were read at 405 nm using the FLUOstar Omega microplate reader (BMG Labtech, Ortenberg, Germany).

### 2.5 Statistical analysis

Statistical analysis was conducted using GraphPad Prism v. 9.1.0 (GraphPad Software, San Diego, CA, USA). Data were checked for normality by Q‐Q plots. For results obtained by BCA and NTA, groups were compared by one‐way ANOVA followed by the Tukey multiple comparison test. Data acquired in the functional studies were compared by two‐way ANOVA (independent variables being *EV‐stimulation* and *LPS‐stimulation*) and further tested by the Tukey multiple comparison test. *p*‐values <0.05 was considered statistically significant. Data are expressed as means ± SD.

## 3 RESULTS

### 3.1 Establishing improved in vitro incubation of *Ascaris suum*

Helminths are complex organisms requiring a host for long‐term survival and completion of their lifecycle. However, they can be incubated ex vivo for a limited time under conditions unique to specific helminths making standardisation across species problematic. To improve methods and pave the way for future guidelines, we optimised several essential aspects of adult *A. suum* incubation from that previously described by Hansen et al. (2019) including pre‐incubation washing, use of different antimicrobials and number of incubation days. The parameters for success were minimal contamination from bacteria and high worm viability. We determined the ideal concentration of an antibiotic‐antimycotic mixture to be 200 μg/ml Streptomycin, 200 U/ml Penicillin G, 1.25 μg/ml Amphotericin B from *Streptomyces sp*. and 10 μg/ml Ciprofloxacin. This combination resulted in a ∼90% decrease in endotoxin levels of the EV preparations and no detection of aerobic bacterial growth on agar plates (Table S1). Including ciprofloxacin during incubation was essential to avoid mycoplasma contamination, which is of particular importance as these bacteria are isolated in the same fractions as EVs (Ridolfi et al., 2020). Lastly, we aimed to determine the longevity of *A. suum* in culture and investigate when conditions were detrimental, which in turn could affect the quality and/or quantity of released EVs. On day four, worms started to show signs of low motility and partial necrosis, indicating distress and reduced viability. Therefore, incubation for a maximum of three days was used in this study. Pilot studies showed that EVs separated from different incubation days showed the same reduction in LPS‐induced TNF release from a human monocytic cell line (THP‐1 cells) (Figure S1). Therefore, EVs released from all three days were pooled for further analyses. The improved conditions for the incubation of adult *A. suum* resulted in ESPs that were consistently negative for mycoplasma and aerobic bacterial growth.

### 3.2 All three separation methods obtain intact EV‐like structures

**FIGURE 2:**
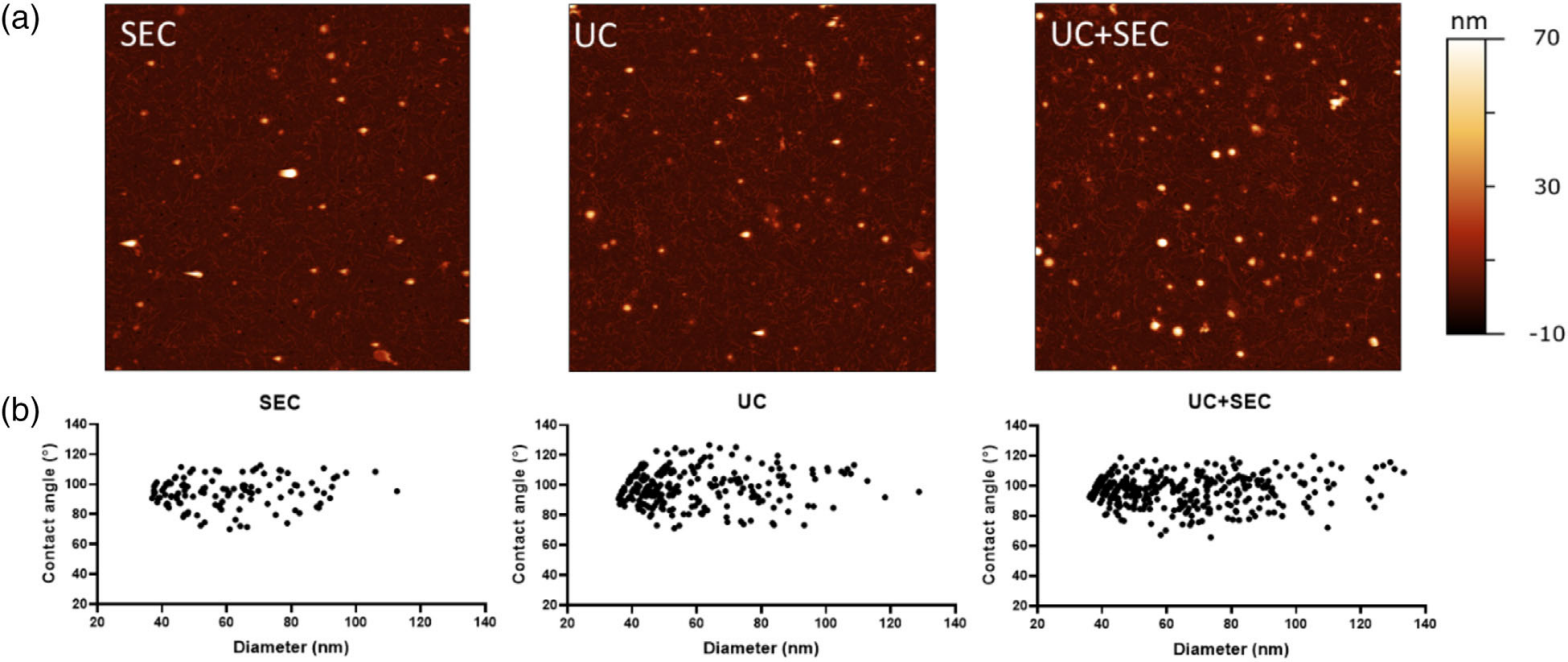
Vesicle‐like structures are identified in all three preparations. (a) Representative AFM images of *Ascaris suum* EVs separated with either size exclusion chromatography (SEC), ultracentrifugation (UC), or a combination of those (UC+SEC). All AFM images are 5 × 5 μm. (b) Contact angle and equivalent diameter of *Ascaris suum* EVs obtained from AFM presented in scatterplots for each EV separation method

The morphology and nanomechanical characteristics of EVs were evaluated using a high‐throughput AFM imaging approach described elsewhere (Ridolfi et al., 2020). Intact EV‐like vesicular structures were identified using all separation methods (Figure 2a). EV‐populations displayed similar size distributions (40–120 nm, average diameter = 60 ± 20 nm, *n* = 642 individual EVs) and a narrow range of vesicle/surface contact angles (average contact angle = 96° ± 9°, *n* = 642) (Figure 2b), corresponding to the typical mechanical stiffness previously reported for *A. suum* EVs (Ridolfi et al., 2020). Aside from identified EVs, smaller globular objects (⌀ = 10–30 nm) which lacked the mechanical signature of intact EVs were also observed. Furthermore, fibrillar fragments with a diameter of 5.6 ± 1.5 nm and lengths of several hundreds of nanometers were also observed in preparation using all separation methods. These fibrils showed a distinct right‐handed helicity (30 ± 4 nm periodicity).

For AFM, all EV preparations were deposited on positively charged substrates following the same protocol, nonetheless resulting in different surface densities of adhered EVs for the different separation methods. In particular, the procedures entailing an UC step (UC and UC+SEC) resulted in roughly 200% more EVs adhering to the substrate compared with the procedure where SEC alone was used, suggesting that UC yields comparatively more concentrated EV samples (Figure 2). These observations demonstrate that combining UC+SEC results in slightly higher EV abundance compared to each separation method alone.

### 3.3 The three separation methods result in similar particle and protein concentration

**FIGURE 3:**
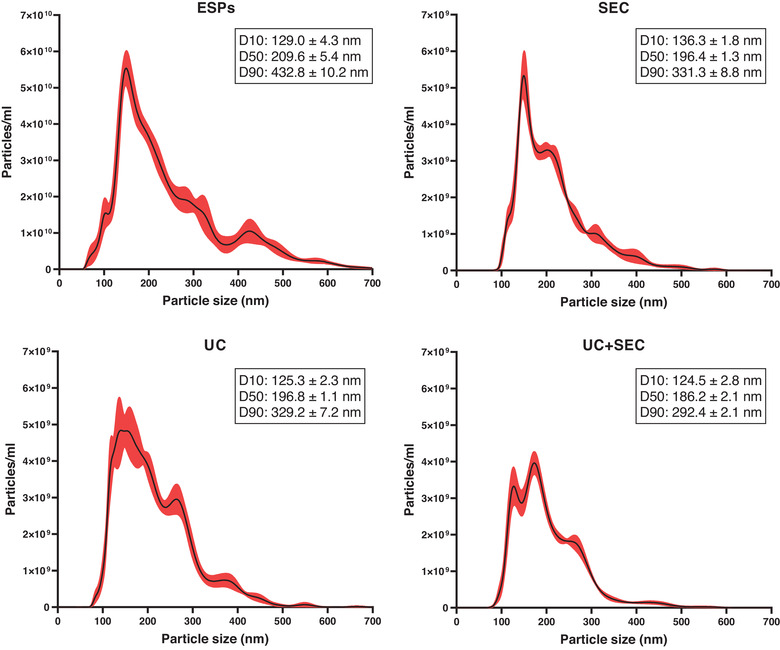
Particle size distribution of the *Ascaris suum* excretory/secretory products (ESPs) and EVs separated from ESPs using size exclusion chromatography (SEC), ultracentrifugation (UC) or a combination of both (UC+SEC). The plots show the average concentration and size of particles based on 5 × 60 sec videos per sample type. The mean concentration is the black line accompanied by a standard error of the mean (red). The inserted boxes show the D10, D50, and D90 corresponding to the percentage of particles below the estimated particle size. Measurements were performed using nanoparticle tracking analysis

**FIGURE 4:**
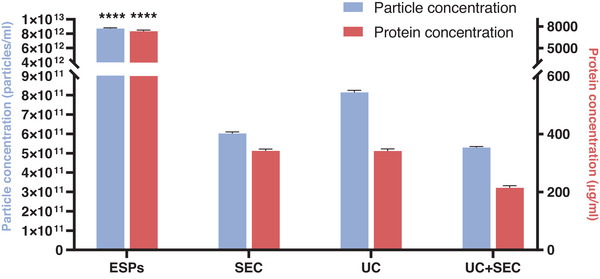
Particle and protein concentration of each separation method. Particle (blue) and protein (orange) quantification of *Ascaris suum* excretory/secretory products (ESPs) and *A. suum* EVs separated using either size exclusion chromatography (SEC), ultracentrifugation (UC), or a combination of both (UC+SEC). Both particle and protein concentrations of ESPs were significantly different compared to all three EV preparations. Groups were compared by one‐way ANOVA followed by a Tukey test. ****p < 0.0001. Error bars: Mean ± SD. *n* = 3 replicates for protein concentration, *n* = 5 replicates for particle concentration

The size and enumeration of EVs were analysed using nanoparticle tracking analysis (NTA) and the mode size was found to be within a range of approx. 140–165 nm regardless of the separation method used. Furthermore, the size distribution profiles in Figure 3 show that SEC‐EVs displayed a narrower size distribution (peaking at 150 nm) of particles compared to UC‐EVs and UC+SEC EVs that presented with broader peaks in the size distribution profile. The application of UC for EV separation yielded the highest concentration of particles followed by SEC and then UC+SEC, however, the differences were not significant (Figure 4). In contrast, ESPs showed a significantly higher concentration of particles compared to the separation procedures (*p* < 0.0001). All three separation methods yield one order of magnitude fewer particles compared to the input material (ESPs). All separation methods resulted in ∼90% loss of particles when compared to the starting ESPs.

The protein concentration (measured by BCA assay) for EVs separated by UC and SEC were similar (341 ± 7 and 342 ± 6 μg/ml, respectively) while the combination of UC and SEC resulted in a ∼1.5‐fold lower concentration (214 ± 7 μg/ml) although the difference was not statistically significant (*p* > 0.05) (Figure 4). Only the ESPs start product was significantly higher in protein concentrated than the other groups (7343 ± 169 μg/ml, *p* < 0.0001). The separation procedures, therefore, resulted in the removal of 95–97% of the proteins measured in the starting material ESP.

### 3.4 Similar protein profiles were obtained between the EV preparations

**FIGURE 5:**
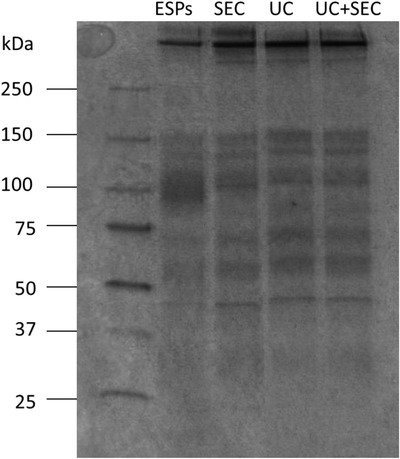
Similar protein profiles between the three separation methods. Silver stain protein profiles of denatured *Ascaris suum* excretory/secretory products (ESPs) and *A. suum* EVs separated with either size exclusion chromatography (SEC), ultracentrifugation (UC) or a combination of both (UC+SEC). For ESPs and SEC, 800 ng protein was loaded into the gel. For UC and UC+SEC, 400 ng protein was loaded into the gel

Due to the lack of verified EV protein markers for helminth EVs, a silver stain was included to compare the protein profiles between separation methods. The silver stain (Figure 5) showed no visible difference in the protein profiles between the three separation procedures. The same bands were observed for all three separation methods, however, they were more separated compared to the ESPs. Bands around 50 and 100 kDa are more intense for EVs from all separation procedures compared to the ESPs. The 100 kDa band is smeared in the ESPs, but clearer in the EV preparations. A single band at above 250 kDa was only observed in the EV preparations.

### 3.5 The combination of UC and SEC obtain the lowest degree of contamination

Most helminth species including *A. suum* live in the intestinal tract making bacterial contamination a potential issue for in vitro incubation. Broad‐spectrum antibiotics during the comprehensive washing steps (*n* = 7 over 4 h) and the 3‐day incubation were introduced to prevent contamination. The ESPs and separated EVs were tested for endotoxin contamination. The highest concentration of endotoxin was found in undiluted EVs separated using SEC (13 EU/ml), followed by EVs obtained by the UC+SEC method (6 EU/ml), while EVs from UC resulted in the lowest endotoxin concentration (3 EU/ml) (Table S1). For the *in vitro* dose‐response experiments (see Section 3.6), the final endotoxin concentration was in the range of 0.3–1.3 EU/ml.

The presence of protein contaminants was evaluated by a multiplex approach using the CONAN assay (Maiolo et al., 2015), particle/protein ratio and AFM analysis.

**FIGURE 6:**
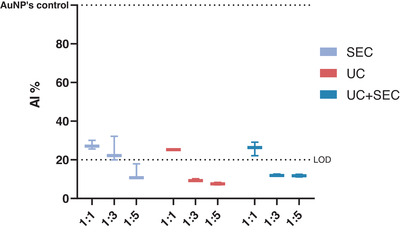
All three separation methods contain a low amount of non‐EV protein contamination. Aggregation index (AI%) ratios of *Ascaris suum* EVs separated using either size exclusion chromatography (SEC), ultracentrifugation (UC) or a combination of those (UC+SEC). The AuNPs control is the AI of monodispersed AuNPs, normalised at 100 and used as a reference. The dotted line (Limit of Detection, LOD) defines the threshold of the CONAN assay for detecting non‐EV protein contamination in the sample (<20% AI means that the protein concentration is ≤0.05 μg/μl). The CONAN assay showed all EV preparations contain low levels of protein contamination. Error bars: Mean ± SD. *n* = 3 replicates

For the CONAN assay EV preparations were analysed as described in Zendrini et al. (2020). The mean aggregation index percentage (AI%) is a ratio between the AI of the EV preparations and the AI of pure monodispersed gold nanoparticles (AuNPs) (normalised to 100). Figure 6 shows that a dilution series of all the preparations resulted in a decreasing AI%. UC and UC+SEC separation methods present an AI% lower than 20% at a dilution of 1:3 with Milli‐Q H_2_O while at a dilution of 1:5 all three preparations showed an AI% lower than 20%. This indicates that all three preparations contained negligible amounts of non‐EV protein contamination.

**FIGURE 7:**
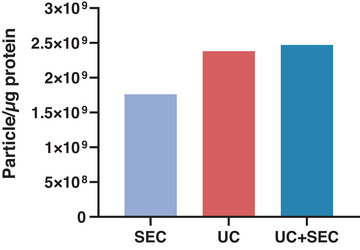
Particle to protein concentration (μg) ratio as an estimate of the purity of *Ascaris suum* EV preparations. The combination of ultracentrifugation (UC) and size exclusion chromatography (SEC) (UC+SEC) resulted in the highest ratio (2.47 × 10^9^ particle/μg protein) suggesting that this EV sample has the lowest protein contamination

To further evaluate the purity of the EV separations a ratio between the concentration of particles and the protein concentration obtained by NTA and BCA was calculated (Webber & Clayton, 2013). The highest particle/protein ratio was obtained by procedures incorporating UC (UC: 2.38 × 10^9^ particle/μg protein, UC+SEC: 2.47 × 10^9^ particle/μg protein), whilst the lowest was the SEC procedure alone (1.76 × 10^9^ particle/μg protein) (Figure 7).

**FIGURE 8:**
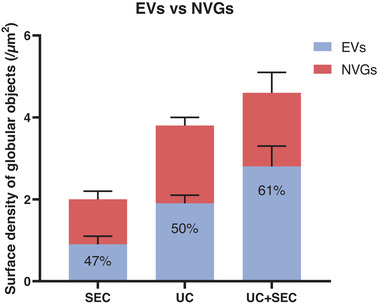
The combination of UC and SEC gave the highest percentage of EVs compared to NVGs. Quantification of EVs (blue) versus non‐vesicular globules (NVGs) (red) in the total amount of objects detected with AFM imaging. EVs separated using size exclusion chromatography (SEC), ultracentrifugation (UC) or a combination of both (UC+SEC) was analysed. The amount of vesicular‐like structures in the EV preparations is stated in percentage. Error bars: Mean ± SD. *n* = 6 for each sample

As a novel approach, AFM can be used to discriminate between EV‐like structures and non‐vesicular globules (NVGs). From AFM images (Figure 2a) it is possible to estimate all deposited spherical shapes as the surface density of globular objects (/μm^2^). Based on the geometrical and mechanical characteristics of these objects, they can be divided into EVs and NVGs. NVGs represented 53% and 50% of all observed objects in SEC and UC preparations, respectively, while their relative abundance dropped to 39% in UC+SEC preparations (Figure 8).

In conclusion, the three methods for estimating the degree of contamination show that despite all three separation methods obtaining a similar yield, the UC+SEC samples present a higher abundance of EVs and lower levels of contamination. The UC method alone results in the lowest amount of endotoxin carry‐over.

### 3.6 *Ascaris suum* EVs reduce LPS‐induced TNF‐α in macrophages and PBMCs in a dose‐dependent manner

The immunomodulatory properties of *A. suum* EVs were assessed in both a monocytic cell line (THP‐1 cells, PMA‐differentiated macrophages) and primary human PBMCs. In both cases, cells were first stimulated with increasing numbers of *A. suum* EVs (particles/cell) and then challenged with LPS in order to induce an inflammatory cell state for the EVs to suppress. Subsequently, the levels of the pro‐inflammatory cytokine TNF‐α release by the cells upon stimuli in the culture media were analysed. Cell viability was not affected by EV‐ and/or LPS‐stimulation (data not shown).

**FIGURE 9:**
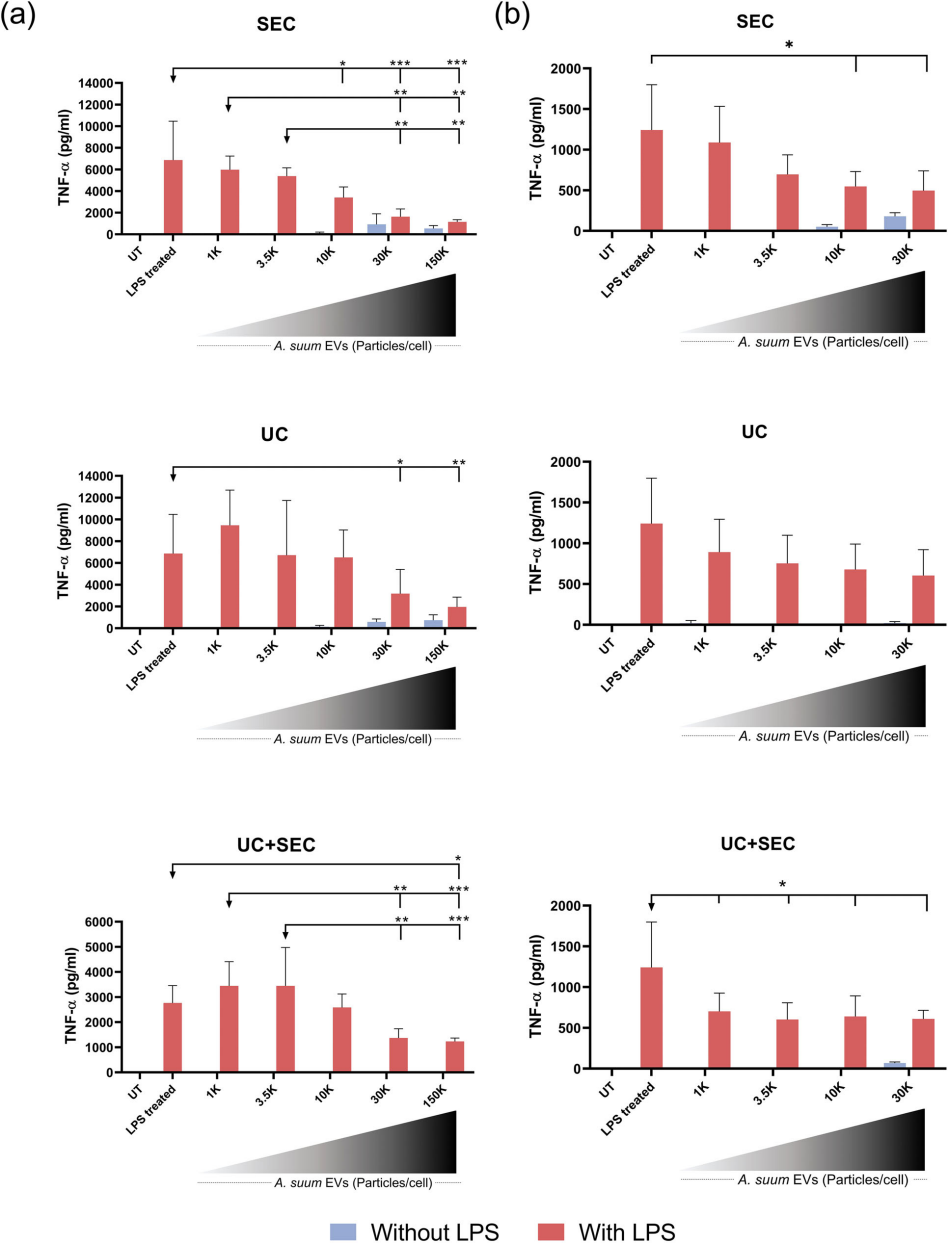
All separation methods resulted in immunomodulatory EVs. (a) TNF‐α release from PMA‐differentiated macrophages (THP‐1 monocytes) or (b) PBMCs 24 h after stimulation with increasing numbers (particles/cell: 1000, 3500, 10,000, 30,000 and 150,000) of *Ascaris suum* EVs (without LPS, blue bars) and subsequent stimulation with 10 ng/ml LPS (with LPS, red bars). The EVs were separated by either size exclusion chromatography (SEC), ultracentrifugation (UC) or a combination of these (UC+SEC). The untreated (UT) is culture media alone and the LPS treated is LPS stimulation alone. Groups were compared using a two‐way ANOVA (independent variables: EV‐stimulation and LPS‐stimulation) followed by a Tukey test. *p < 0.01, **p < 0.01, ***p < 0.001. Error bars: Mean ± SD. *n* = 3 replicates for THP‐1 cells, *n* = 3 donors in duplicates for PBMCs

LPS‐induced TNF‐α levels released by THP‐1 macrophages were reduced in a dose‐dependent manner by EVs compared to LPS alone independent of the EV separation method used (Figure 9a). The same effect was observed for stimulation of PBMCs (Figure 9b). When given at the highest concentration tested, EVs induced TNF‐α release in the absence of LPS. EV‐depleted fractions did not reduce LPS‐induced TNF‐α (Figure S2). Surprisingly, we found that *A. suum* EVs were unable to reduce the LPS‐induced production of IL‐1β in PBMCs (Figure S3).

In conclusion, stimulation of THP‐1 macrophages and PBMCs with *A. suum* derived EVs resulted in a comparable dose‐dependent reduction of LPS‐induced TNF‐α release while a high number of EVs (particles/cell) independently may induce a pro‐inflammatory response. The effects were independent of the EV separation method applied.

## 4 DISCUSSION

In light of the recent discovery of helminth‐derived EVs, a plethora of opportunities has risen for their diagnostic and therapeutic potential. By elucidating EV mediated mechanisms of host immune response modulation, helminth EVs could improve our understanding of host‐parasite interactions. However, a prerequisite for investigating helminth EVs is appropriate ex vivo culturing conditions to obtain ESPs for subsequent EV production. We focused on ex vivo conditions that minimised endotoxin contamination in ESPs whilst keeping helminths viable. Furthermore, we compared three commonly used separation procedures for their efficiency in obtaining helminth EVs with low levels of protein and endotoxin contamination whilst maintaining high yield and retaining EV functional properties. The separation methods were assessed by state‐of‐the‐art characterisation methods according to the MISEV 2018 guidelines (Théry et al., 2018) for EVs as well as evaluation of their functional properties in vitro.

We used a high throughput AFM‐based approach for detailed morphological characterisation of EVs from each separation method. The EVs were in the size range of 40–120 nm in all EV preparations, which is smaller than the size measurements obtained by NTA (140–165 nm). NTA measures the hydrodynamic diameter of particles, which is always larger than their geometrical diameter, as previously discussed by Bachurski et al. (2019). Furthermore, the sensitivity of the Nanosight system is limited to minimal detectable sizes of 70–90 nm for biological vesicles and therefore is unable to identify smaller vesicles that can be identified by AFM (Van Der Pol et al., 2014). The size distribution profiles estimated using NTA showed the narrowest size profile for EVs separated using SEC whereas particles from UC and UC+SEC displayed a broader size distribution. Separating mammalian EVs with UC has previously been shown to be accompanied by the potential introduction of EV aggregates, which might explain the wider size distribution observed with this method (Linares et al., 2015). This tendency was also found in other comparative studies (Brennan et al., 2020; Hansen et al., 2019).

The UC method resulted in the highest particle concentration followed by SEC and UC+SEC. This is in contrast to mammalian EVs where the SEC method resulted in the separation of the highest number of particles (Askeland et al., 2020; Brennan et al., 2020; Takov et al., 2019). However, a study by Mol et al. (2017) found no significant difference in particle yield between UC and SEC, supporting our findings. The different nature of EVs from various sources and the physical properties of these sources may explain this inconsistency (Dong et al., 2020). Since UC separates EVs based mainly on density, EVs from *A. suum* may have a higher density compared to mammalian EVs explaining this difference in the outcome. Furthermore, the precise method of separating EVs with centrifugation (or differential centrifugation) can vary on several parameters (e.g., rotor type, centrifugation speed and time) further making a direct comparison between studies difficult (Stam et al., 2021).

During EV separation there was a ∼90% loss of particle concentration, however, the NTA measures all particles hence also non‐EVs making it a poor estimate for total EV concentration (Bachurski et al., 2019). As an alternative approach, we used AFM imaging to estimate the relative abundance of EVs in the three samples. As a surface technique, AFM cannot directly measure the concentration of EVs in a solution, requiring their deposition on a substrate. However, if the deposition protocol is kept constant, highly concentrated solutions will decorate the substrate with comparatively more objects than those at lower concentrations. In other words, the surface density of globular objects can be used to estimate the relative concentrations of the starting solutions, as shown elsewhere (Caselli et al., 2021). Moreover, AFM can discern EVs from NVGs based on their nanomechanical characteristics, making it possible to compare the relative abundance of true EVs. This approach revealed that EVs were more abundant in samples obtained using the UC+SEC method compared to the SEC and UC methods in separation. This finding is further supported by our observed particle/protein ratios, when used as an estimate of EV purity as suggested by Webber and Clayton (2013). The UC+SEC combination for EV separation resulted in the highest particle/protein ratio (2.47 × 10^9^ particle/μg protein). According to Webber and Clayton (2013), a ratio between 2 × 10^9^‐2 × 10^10^ particle/μg protein equate to *low purity* making the EV preparations of this study unsatisfactory. However, our estimate is similar to that obtained for EVs released by the helminth *Trichuris muris* (highest value 4.31 × 10^9^ particle/μg protein) (Eichenberger et al., 2018). In contrast to our results, Brennan et al. (2020) found SEC to obtain the highest particle/protein ratio for mammalian serum EVs compared to centrifugation‐based separation. The measured proteins in the EV preparation might be EV‐related (as seen in Figure S4) making this purity estimate flawed. Whether the purity of EV preparations can be accurately evaluated by this ratio and whether this method can be applied for helminth EVs is contradicted by the CONAN assay, which showed all EV preparations to have insignificant contamination by non‐EV proteins.

*A. suum* has shown suppression of pro‐inflammatory responses in vitro and in vivo (Almeida et al., 2018; Midttun et al., 2018), however, the contribution of EVs to this effect remains unknown. Hansen et al. (2019) demonstrated the release of EVs from *A. suum* (from all life stages) that contain immunosuppressive miRNAs targeting processes for T‐cell activation and production of cytokines. In this work, we further establish that adult *A. suum* release immunomodulatory EVs. *A. suum* EVs suppressed LPS‐induced TNF‐α production in both a cell line and primary cells in a dose‐dependent manner. Conversely, high numbers of EVs induced TNF‐α by themselves. Whether this effect is related to the EVs or contaminating factors in the preparations is unknown. Importantly, we did not observe any functional differences in this *in vitro* assay between the EVs from the three different separation methods.

Despite the three methods using either size or density for separating EVs, they do not seem to yield distinct subpopulations of EVs, which is in agreement with protein profiles on the silver stain. Unfortunately, few studies have investigated the functionality of EVs separated by different methods. Mol et al. (2017) found SEC to obtain EVs of higher functionality measured by extracellular signal‐regulated kinase1/2 (ERK1/2) phosphorylation compared to UC and suggested that UC results in rupture of EVs. Helminth EVs display a unique glycan and lipid composition compared to mammalian EVs, which might attribute to a more robust construction that is less affected by centrifugation (Whitehead et al., 2020). However, EVs affect the recipient cells in multiple ways making it challenging to elucidate every aspect of their complexity and thus challenging to fully compare between studies.

Several studies have compared different separation methods for mammalian EVs aiming to standardise methodologies, but there is only one comparative study on helminth EVs. Using *Fasciola hepatica* as an EV source, Davis et al. (2019) concluded that SEC is preferred over differential centrifugation, which included UC steps. Based on our results, we only find marginal differences between the methods, but we do find that UC+SEC reduced the number of potential contaminating factors. This discrepancy may relate to the different helminth species used and differences in their EVs composition, but is perhaps more likely due to differences in pre‐clearance procedures and centrifugation settings (Davis et al., 2019). In conclusion, while our comparison of three separation procedures for *A. suum* EVs showed minor variations between EV preparations with regards to characterisation and functionality, our data suggest that combining UC+SEC results in lower protein and NVGs contamination. Further comparative studies on helminth‐derived EVs are highly warranted in order to establish standardisations of separation methods and EV characteristics in the rapidly evolving field of EVs.

## 5 LIMITATIONS TO THE STUDY

The included separation methods were carefully selected based on the most frequently applied (Royo et al., 2020) and their target of unspecific EV properties for separation, that is, size and density. Affinity‐based methods were excluded as these are developed for mammalian EVs and their surface markers, which are as yet unknown for helminth EVs. This highlights another limitation already addressed in the paper, that commonly applied mammalian EV markers are not translatable to helminth EVs.

The experimental setup for testing immunomodulatory properties was limited to testing the immunosuppressive ability of *A. suum* EVs upon LPS stimulation. LPS targets primarily TLR4 (toll‐like receptor 4) that is highly present on myeloid cells such as monocytes (Bertani & Ruiz, 2018; Vaure & Liu, 2014). Other agonists could give different results and thereby investigate other aspects of inflammation. Furthermore, the readout for functional studies was limited to a simple setup with cytokine measurements (only TNF‐α presented in the paper). Expanding the functional readout to include more cytokines or other methods of analysis could further qualify the conclusion. However, as this research was focused upon a technical comparison of EV separation methods, we believe it is beyond the scope of this paper to elaborate more on the functional characteristics.

## DECLARATION OF INTERESTS

The authors declare no competing interests.

## AUTHOR CONTRIBUTIONS

Conceptualisation: Anne Borup and Peter Nejsum; Methodology: Anne Borup, Anders T. Boysen; Andrea Ridolfi, Marco Brucale, Francesco Valle and Lucia Paolini; Validation: Anne Borup; Formal analysis: Anne Borup, Marco Brucale and Lucia Paolini; Investigation: Anne Borup, Anders T. Boysen, Andrea Ridolfi, Marco Brucale and Lucia Paolini; Resources: Francesco Valle, Paolo Bergese and Peter Nejsum; Data curation: Anne Borup; Writing – Original draft: Anne Borup; Writing – Review & editing: Anne Borup, Anders T. Boysen, Andrea Ridolfi, Marco Brucale, Francesco Valle, Lucia Paolini, Paolo Bergese and Peter Nejsum; Visualisation: Anne Borup and Marco Brucale; Supervision: Peter Nejsum; Project administration: Anne Borup; Funding acquisition: Paolo Bergese and Peter Nejsum. All authors have read and agreed to the published version of the manuscript.

## Supporting information

Supporting Information
