# Supplementary material for: Comparison of separation methods for immunomodulatory extracellular vesicles from helminths
Source: J Extracell Biol. 2022 May 3;1(5):e41. doi: 10.1002/jex2.41 (PMC11080882; doi:10.1002/jex2.41)
Supplement: Supplementary file 1 — Supporting Information [file JEX2-1-e41-s001.docx]

Supplementary information

| Sample | Low antibiotics^a^ (EU/ml) | High antibiotics^b^ (EU/ml) |
| --- | --- | --- |
| SEC EVs | 177.7 | 13.3 |
| UC EVs | 117.7 | 3.5 |
| UC+SEC EVs | 67.9 | 5.5 |

**Table S1** **Endotoxin levels (EU/ml) in EV-samples separated with either size exclusion chromatography (SEC), ultracentrifugation (UC) or a combination of both (UC+SEC). Related to STAR Methods.** a = 100 µg/ml Streptomycin, 100 U/ml Penicillin G, 0.63 µg/ml Amphotericin B and 5 µg/ml Ciprofloxacin, b = 200 µg/ml Streptomycin, 200 U/ml Penicillin G, 1.25 µg/ml Amphotericin B and 10 µg/ml Ciprofloxacin.

**Figure S1** **Similar immunomodulatory effect from EVs separated from different helminth incubation days. Related to STAR methods.** TNF-α release from PMA-differentiated macrophages (THP-1 cells) 24 hours after stimulation with increasing numbers (particles/cell) of Ascaris suum EVs (blue bars) and 10 ng/ml LPS (red bars). The EVs were isolated by size exclusion chromatography (SEC) from either day one, three or five of incubation. The untreated (UT) is culture media alone and the LPS treated is LPS stimulation alone. Error bars: Mean ± SD. n = 3 replicates.

**Figure S2 The EV-depleted fraction does not present immunomodulatory effects. Related to Figure 8.** TNF-α release from PMA-differentiated macrophages (THP-1 monocytes) 24 hours after stimulation with increasing concentration (µg/ml) of Ascaris suum EV-depleted fractions (blue bars) and 10 ng/ml LPS (red bars). The EV-depleted fractions were created by either size exclusion chromatography (SEC) or ultracentrifugation (UC). For SEC, the EV-depleted fractions were 12-24, while for UC, it was the supernatant. Excretory/secretory products (ESPs) were included to compare to the starting material. The untreated (UT) is culture media alone and the LPS treated is LPS stimulation alone. Groups were compared using a two-way ANOVA (independent variables: EV-stimulation and LPS-stimulation) followed by a Tukey test. *** = p < 0.001. Error bars: Mean ± SD. n = 3 replicates.

**Figure S3 Levels of LPS-induced IL-1β is unaffected by EV-stimulations. Related to Figure 8.** IL-1β release from PBMCs 24 hours after stimulation with increasing numbers (particles/cell: 1000, 3500, 10,000, 30,000 and 150,000) of Ascaris suum EVs (without LPS, blue bars) and subsequent stimulation with 10 ng/ml LPS (with LPS, red bars). The EVs were separated by either size exclusion chromatography (SEC), ultracentrifugation (UC) or a combination of these (UC+SEC). The untreated (UT) is culture media alone and the LPS treated is LPS stimulation alone. Error bars: Mean ± SD. n = 3 donors in duplicate.

**Figure S4 Particle and protein concentration for each fraction generated by SEC. Related to STAR Methods.** Particle (blue) and protein (orange) concentration of Ascaris suum excretory/secretory products (ESPs) and the 24 fractions collected from size exclusion chromatography (SEC). The fractions 7-10 was identified as the EV enriched fractions. Error bars: Error bars: Mean ± SD. n = 5 replicates for particle concentration, n = 3 replicates for protein concentration.
